# Association of *interleukin-17F* (rs763780) single nucleotide polymorphism with multiple sclerosis and optic neuritis

**DOI:** 10.1038/s41598-024-62736-2

**Published:** 2024-06-13

**Authors:** Shereen Salah, Yousra I. Sadeq, Youssef M. Mosaad, Ibrahim E. H. Elmenshawi, Ziyad M. E. Tawhid

**Affiliations:** 1 Clinical Immunology Unit, Clinical Pathology Department, Faculty of Medicine, Mansoura University, Mansoura, Egypt; 2 Clinical Immunology Unit, Clinical Pathology Department and Mansoura Research Center for Cord Stem Cells (MARC_CSC), Faculty of Medicine, Mansoura University, Mansoura, 35111 Egypt; 3 Neurology Department, Mansoura Faculty of Medicine, Mansoura University, Mansoura, Egypt

**Keywords:** IL-17F, SNP, MS, RT-PCR, Egyptian, Genetics, Immunology, Molecular biology, Neurology

## Abstract

*IL-17F* single nucleotide polymorphism (SNP) can affect IL-17F expression and activity and this can lead to the increased susceptibility to several autoimmune diseases. The aim was to investigate the association of *IL-17F* (rs763780) SNP with the development of multiple sclerosis (MS) in a cohort of Egyptian patients and to evaluate the effect of this polymorphism on the disease course. *IL-17F* (rs763780) gene polymorphisms *was* typed by TaqMan genotyping assay for 231 Egyptians divided into 102 MS patients and 129 healthy controls with matched age and sex. The *IL-17F* rs763780* C* containing genotypes (CT+CC) and C allele have statistically significant increased frequency in MS patients when compared with controls (*p* = 0.005 and 0.004 respectively) especially in females’ patients (*p* = 0.005 and 0.006 respectively). The heterozygous CT genotype was associated with the presence of optic neuritis (*p* = 0.038). The multivariable regression analysis revealed significant associations between smoking, the higher frequency of attacks and the prediction of higher EDSS score (*p* = 0.032, 0.049 respectively). It can be concluded that the IL-17F rs763780 C containing genotypes (CT and CC) and C allele may be risk factors for the development of MS in the studied Egyptian cohort by a gender-dependent mechanism that contributes to tendency for predisposition in females and optic neuritis is more common in patients carrying the CT heterozygous genotype.

## Introduction

Multiple sclerosis (MS), is a chronic autoimmune neuro-inflammatory disorder of the central nervous system, and is characterized by multiple foci of demyelination of the CNS due to damaging of the insulating covers of nerve cells in the brain and spinal cord^1^. MS is the commonest disabling non-traumatic disease that affects young adults^2^. The incidence and prevalence of MS is increasing in all over the world^3^. The underlying cause still unclear and is complex due to the involvement of increasing number of susceptibility genes and several environmental factors such as vitamin D, exposure to ultraviolet rays, obesity, smoking and infection by Epstein–Barr virus (EBV)^4^.

MS is now classified as an autoimmune disease and is mediated by the involvement of many aspects of activated immune system including activation of T lymphocytes, activation of B lymphocytes and activation of innate immune system cells^5^. MS patients are classified into four major categories according to the course of the disease: relapsing remitting MS, secondary progressive MS, primary progressive MS, and progressive-relapsing MS^6^*.*

The Th17 cells are the main source of IL-17 cytokine family which includes six members from IL-17A to IL-17F. IL-17 cytokines are immediately released and contribute to epithelial homeostasis, inflammatory acute responses and B cell stimulation^6,7^, thus acting as a bridge between innate and acquired immune responses. IL-17 increased expression was found in the autopsy of brain patients with MS, and in active CNS lesions, abundant IL-17-expressing cells were found^6–8^. *IL-17F* gene is localized on chromosome 6p12 and is co-expressed by Th17 and γδ T cells. The* IL-17F* rs763780 SNP, a His-to-Arg substitution at amino acid 161 (H161R) in the third exon of the *IL17F* gene, enhances the expression and activity of IL-17F^9^, decreases the ability of IL17F to induce expression of certain cytokines and chemokines^10^, and binds to the receptors of the wild-type, hence, acts as a natural antagonist of the wild-type^10–12^. The associations were reported between *IL17F* rs763780 SNP and several inflammatory, autoimmune, infectious disorders, and even cancers^10–12^.

The susceptibility to MS was investigated in the genome-wide association study of International Multiple Sclerosis Genetics Consortium (IMSGC) and they identified association between MS and more than 150 single nucleotide polymorphisms (SNPs)^13^. However, small odds ratio was found in most of the SNPs and the chromosomal position of many of these SNPs were close to genes associated with immune function^4,13^.

The polymorphisms of IL-17F have been found to be associated with the increased susceptibility to different autoimmune diseases such as rheumatoid arthritis^14^, ulcerative colitis^15^ and multiple sclerosis^16^. Maria et al.^7^ comprehensive review discussed the most recent data related to the use of IL-17A blocking agents in the treatment of different immune (rheumatoid arthritis), inflammatory (inflammatory bowel disease), cardiovascular, hepatic, cancers either hematologic or solid^7^. Movement of activated Th17 from periphery into the CNS occurs early in MS and the use of Natalizumab in the treatment of severe RR MS was associated with blocking of such trafficking and resulted in peripheral accumulation of Th17^7,17^. Therefore, studying the association between the IL-17F gene polymorphism and MS in Egyptians may help in early diagnosis, prevention, and treatment. The present study was aimed to investigate the association between IL-17F rs763780 SNP and the development of MS in a cohort of Egyptian patients and to evaluate its effect on the disease course.

## Materials and methods

This is a case control study that was conducted on 231 individuals (102 MS patients and 129 apparently healthy control subjects) matched in age and sex. The patients age ranged from 18 to 54 years with (mean ± SD = 32.3 ± 8 y) with 41 males and 61 females. The control age ranged from 17 to 54 years with (mean ± SD = 31.3 ± 4.7 y), with 53 males and 76 females. All patients gave informed consent to their participation in this study, and the research was approved by the institutional research board (IRB) under number (MD/169).

MS was diagnosed according to McDonald 2017 criteria for Diagnosis of MS^18^. The patient was subjected to full history and complete neurological examination with special emphasis on the presence of multiple sclerosis-associated symptoms (mobility problems, vision problems, problems with coordination, cognitive dysfunction, fatigue, and pain), as well as duration of the disease. All patients were subjected to Magnetic resonance imaging (MRI) to confirm the diagnosis of MS.

The MS disease course was classified into four major categories^1^. Relapsing–remitting MS (RRMS), it is marked by flare-ups (relapses or exacerbations) of symptoms followed by periods of remission when symptoms improve or disappear. Secondary progressive MS (SPMS), SPMS follows an initial RRMS and characterized by progressive worsening of neurologic function (accumulation of disability) over time. Primary progressive MS (PPMS) is characterized by worsening neurologic function (accumulation of disability) from the onset of symptoms with no relapses or remissions. Progressive-relapsing MS (PRMS) is progressive from the start, with intermittent flare-ups of worsening symptoms along the way and with no periods of remission.

Determination of expanded disability status scale (EDSS) score was done to measure the severity of MS. The EDSS score ranges from 0 to 10 in 0.5-unit increments. A score of 0 means no signs or symptoms; score of ≤ 3 represent mild disability with no or minimal impairment of ambulation, score of 3 to 6 refer to moderate to relatively severe disability and impairment of gait and daily routine but patient still able to walk; score of ≥ 6 refers to more severe disability and requiring assistance with walking, while an EDSS of 10 refers to death related to MS^19^. Exclusion criteria of cases were history of chronic CNS diseases, other autoimmune diseases, Children and refused patients.

Two and half ml of venous blood were collected from all controls and patients by clean venipuncture using plastic disposable syringes then delivered into 5 ml sterile vacutainer tubes containing 50 µl tri-potassium EDTA solutions and stored at − 20 °C until DNA extraction was done.

### Genotyping of *IL-17F* rs763780

The studied SNP comprised of C and T alleles. It is located on chromosome 6 on IL17F gene. Allele change from CAT into CGT leads to amino acid change from histidine into arginine. Table 1 Genomic DNA was extracted from whole venous EDTA blood using Thermo Scientific Gene JET whole Blood Genomic DNA Purification Mini Kits. DNA concentration was measured by NanoDrop 2000c (thermo Scientific, USA). Then the extracted DNA samples were stored at − 20 °C until use.

**Table 1:** Genetic characteristics of studied polymorphism.

ID	rs763780
Alleles	C/T
Ancestral Allele	T
Chromosomal location	6p12.2
Gene	IL17F; interleukin 17F
Gene family	Interleukins
Allele change	CAT ⇒ CGT
Amino acid change	H [His] ⇒ R [Arg]

### TaqMan SNP genotyping assays (real time PCR)

Genotyping was done by real-time PCR using TaqMan SNP genotyping assay technique (Applied Biosystem, lot 4440042, Foster, USA). The SNP ID is C_2234166_10 for *IL-17F* rs763780 and the chromosomal location is Chr.6: 52101739. One probe labeled with VIC^®^ dye detects the Allele C sequence & the other probe labeled with FAM™ dye detects the Allele T sequence. The context Sequence [VIC/FAM] was GTG GAT ATG CAC CTC TTA CTG CAC A[C/T] GGT GGA TGA CAG GGG TGA CGC AGG T. Twenty microliter of PCR reaction mix for qPCR analysis was prepared as follow: 10.0 ul TaqMan^®^ universal master mix II with UNG, 2X (Applied Biosystem, lot 4440042), 1.0 ul TaqMan^®^ assay 20X and 5.0 µl DNA template + 4 ul RNase-free water. For each sample, twenty ul of PCR reaction mix was transferred to the 48-well Reaction plate. The plate was sealed using appropriate cover and was briefly centrifuged and loaded into the real-time PCR (Step one, Applied Biosystem, USA). Reaction conditions were carried out with the following cycling stages; stage I for UNG incubation at 60 °C for 30 s; stage II for polymerase activation at 95 °C for 10 min; stage III for PCR and includes 50 cycles of denaturation at 95 °C for 15 s and annealing/extension at 60 °C for 90 s and finally stage IV 60 °C for 30 s. Allelic discrimination was carried out by measuring fluorescence intensity at the endpoint. The SDS software version 1.7 (Applied Biosystem, Foster, USA) was used for typing of genotypes and alleles of the SNPs. Each sample was interpreted according to the increase in fluorescence intensity of either FAM (TT homozygous genotype) or VIC (CC homozygous genotype) or both FAM and VIC (CT heterozygous genotype). Ten percent of samples were repeated for confirmation.

#### Statistical analysis

The statistical analysis of data was done using excel program (Microsoft Office 2013) and IBM SPSS (statistical package for social science) program (SPSS, Inc, Chicago, IL) version 20. Qualitative data were presented as frequency and percentage. Chi square and Fisher’s exact tests were used to compare groups. Quantitative data were presented by mean, ± SD, median and range. Comparisons between two groups were done using t-test or Man Whitney (for non-parametric). Deviations from Hardy–Weinberg equilibrium expectations were determined using the chi-square test Goodness-of-fit test. Both cases and control groups were found to be in agreement with Hardy Weinberg equilibrium. Linear regression analysis was computed using generalized linear model. Odds ratio and 95% confidence interval were calculated. N.B: *p* is significant if < 0.05 at confidence interval 95%.

### Ethical approval and consent to participate

The study was approved by the local Ethical Committee: Institutional Research Board (IRB) of Mansoura Faculty of Medicine (MD/169) and All methods were performed in accordance with the ethical standards as laid down in the Declaration of Helsinki and its later amendments or comparable ethical standards. Written informed consent was obtained from all participants from a parent and/or legal guardian.

## Results

The clinical characteristics of MS patients are presented in Table 2. The distribution of *IL-17F* rs763780 genotype and allele frequencies showed significantly higher frequency of TT genotype (69% vs. 51%, *p* = 0.005), and T allele (83.7% vs. 72.5%, *p* = 0.004) among control group. In MS patients, a significant increased frequency was observed for the CT (43.1% vs. 29.5%, *p* = 0.015), CC (5.9% vs. 1.6%, *p* = 0.045), CT+CC (49% vs. 31.1%, *p* = 0.005) genotypes and for the C allele (27.5% vs. 16.3%, *p* = 0.004) Table 3. At the same time, the distribution of the genotypes and alleles of *IL-17F* rs763780 was analyzed in patients and controls based on gender. In comparison with controls, the female patients had statistically significant higher frequency of CT, CT+CC genotypes and C allele (*p* = 0.008, 0.005 and 0.006 respectively). Table 4

**Table 2:** Clinical characteristics of MS patients.

Parameters	MS patients (no = 102)	
Age years (M ± SD)	32.3	8.0
Male (N/%)	41	40.2
Female (N/%)	61	59.8
MS clinical course (N/%)		
Relapsing remittent MS (RRMS)	82	80.4
Secondary progressive MS (SPMS)	20	19.6
EDSS (Median/range)	2.5	1–8
EDSS score (N/%)		
≤ 3	63	61.8
3–6	25	24.5
≥ 6	14	13.7
Total no. of attacks (Median/range)	2.0	1–25
Duration of disease (years) (Median/range)	4.0	0.1–19
Age at disease onset (Median/range)	26.0	14–54
Smoking (Yes/No) (N/%)	84/18	82.4/17.6
Optic neuritis (yes/No) (N/%)	63/39	61.8/38.2
Optic neuritis (n = 63)		
Unilateral /bilateral (N/%)	37/26	58.7/41.3

**Table 3:** Distribution of IL-17F rs763780 genotypes and alleles in MS patients and healthy controls.

rs763780		Control (n = 129)		MS (n = 102)		OR	95% CI	*P* value
		N	%	N	%			
Genotype	TT	89	69	52	51	0.467	0.273–0.801	0.005*
	CT	38	29.5	44	43.1	1.532	1.086–2.162	0.015*
	CC	2	1.6	6	5.9	2.744	1.043–7.222	0.041*
	TC+CC	40	31	50	49	1.608	1.150–2.247	0.005*
HWE		0.36		0.4		–	–	–
Allele	T	216	83.7	148	72.5	0.514	0.327–0.807	0.004*
	C	42	16.3	56	27.5	1.946	1.239–3.056	0.004*

*OR* odds ratio, *CI* confidence intervals, *HWE* Hardy Weinberg equation.

*Significant (*P* value < 0.05).

**Table 4:** Distribution of IL-17F (rs763780) genotypes and alleles regarding gender in MS patients and healthy control subjects.

IL-17F (rs763780)			MS Patients N = 102		Controls N = 129		OR	95% CI	*P* value
Males	Genotype	Total	N = 41	%	N = 53	%			
		TT	25	61.0	37	69.8	0.676	0.27–1.60	0.371
		CT	13	31.7	15	28.3	1.168	0.66–2.05	0.588
		CC	3	7.3	1	1.9	2.508	0.64–9.90	0.189
		CT+CC	16	39.0	16	30.2	1.278	0.75–2.19	0.371
	Allele	T	63	76.8	89	84.0	0.633	0.31–1.31	0.218
		C	19	23.2	17	16.0	1.579	0.76–3.28	0.218
Females	Genotype	Total	N = 61	%	N = 76	%			
		TT	27	44.3	52	68.4	0.367	0.18–0.74	0.005*
		CT	31	50.8	23	30.3	1.812	1.17–2.82	0.008*
		CC	3	4.9	1	1.3	2.951	0.75–11.56	0.120
		CT+CC	34	55.7	24	31.6	1.869	1.21–2.88	0.005*
	Allele	T	85	69.7	127	83.6	0.452	0.25–0.81	0.006*
		C	37	30.3	25	16.4	2.211	1.24–3.94	0.006*

*R* reference, *OR* odds ratio, *CI* confidence intervals.

*Significant (*P* value < 0.05).

The distribution of *IL-17F* rs763780 genotypes was compared in relation to the clinical data of MS patients and non-statistically significant differences were found (*p* > 0.05) except for the significant association between the heterozygous CT genotype and presence of optic neuritis (*p* = 0.038). Table 5 Regression analysis for the prediction of higher EDSS score was done using age, gender, smoking, age of disease onset, duration of disease, total number of attacks and *IL-17F* (rs763780) genotypes as covariates and presented in Table 6. The multivariable analysis revealed significant associations between smoking, and higher frequency of attacks and the prediction of higher EDSS score (*p* = 0.032, 0.049 respectively). However, in multivariable analysis, smoking as well as higher frequency of attacks are considered as bad predictors of higher EDSS score (*p* = 0.032, 0.049 respectively).

**Table 5:** Relation between genotype frequencies of IL-17F rs763780 and clinical data of MS patients.

Data	IL-17F								P1	P2
	TT (N = 52)		CT (N = 44)		CC (N = 6)		CT+CC (n = 50)			
Age of onset years (median/range)	26.8 14–54		26.5 16–49		24 19–43		26 16–49		0.79	0.639
Disease duration years (N/%) (median/range)	3.75 0.1–12		4.0 0.1–19		5.0 2.0–7.0		4.0 0.1–19.0		0.74	0.721
Number of attacks (N/%) (median/range)	2.0 1.0–25		2.0 1.0–25		2.0 2.0–4.0		2.0 1.0–25.0		0.61	0.501
RRMS (N = 82) (N/%)	45	54.9	32	39.0	5.0	6.1	37.0	74.0	0.83	0.111
SPMS (N = 11) (N/%)	7.0	35	12.0	60	1.0	5.0	13.0	26.0		
Optic neuritis (N/%)										
Yes	27	42.9	32	50.7	4.0	6.4	36.0	72.0	0.038*	0.037*
No	25	64.1	12	30.8	2.0	5.1	14.0	28.0		
Optic neuritis (N/%)										
Unilateral	18	48.6	17	46.0	2.0	5.4	19.0	52.8	0.29	0.268
Bilateral	9.0	34.6	15.0	57.7	2.0	7.7	17.0	47.2		
EDSS										
< 3	35	55.6	25	52	3.0	28.6	28.0	56.0	0.89	0.187
3–6	13	39.7	11	44	1.0	57.1	12.0	24.0		
> 6	4.0	4.8	8.0	4.0	2.0	14.3	10.0	20.0		

P1, comparison between TT, TC and CC; p2, comparison between CT+CC versus TT.

**Table 6:** Regression analysis for prediction of EDSS scores.

EDSS	Univariable			Multivariable		
	*P*	OR	95% CI	*P*	OR	95% CI
Age	0.642	0.988	0.937–1.041			
Gender	0.923	1.041	0.459–2.365			
Smoking	0.015*	1.928	1.136–3.273	0.032*	1.794	1.053–3.056
Age of disease onset	0.167	0.964	0.916–1.015			
Duration of disease	0.043*	1.121	1.003–1.251	0.409	1.030	0.960–1.105
Total number of attacks	0.002*	1.175	1.062–1.301	0.049*	1.066	1.001–1.137
CC+CT versus TT	0.187	1.308	0.878–1.947			

*OR* odds ratio, *CI* confidence interval; ordinal regression was used.

*Significant (*P* value < 0.05).

## Discussion

MS is one of the autoimmune chronic neuro-inflammatory demyelinating diseases of the CNS that is a common cause of serious physical disability in young adults, especially women^20^. The MS brain and nerve damage is due to CNS inflammation. Although, the exact etio-pathogenic factor that initiates the inflammation is unknown, however, infectious, genetic, and environmental factors have been suggested^21^. Hashem et al.^22^ meta-analysis reported the prevalence of MS to be 14.1/100,000 in Egyptian and 25/100.000 by El-Sawy et al.^23^.

While T helper 1 (Th1) proinflammatory cytokines, IFN-γ, TNF-α, IL-1, IL-2, and IL-12, have been linked to the onset and perpetuation of MS, the Th2 anti-inflammatory cytokines, IL-4, IL-10 and TGF-β, have been linked to MS remission^24^. It was known that the MS disease is a Th1-cell-driven. Earlier, Th17 cells have been considered as important players in the immune pathogenesis of MS^25^. IL-17 transcription is up-regulated in the MS lesion^26^, IL-17F levels are elevated in the serum and CSF from patients with active MS^27^ and memory T cells producing IL-17 infiltrate into MS lesions^28^. IL-17F, a member of IL-17 family, is a pro-inflammatory cytokine and induces the expression of various cytokines, chemokines, and adhesion molecules. Therefore, it is proved to have a crucial role in the emergence of autoimmune diseases^29^.

Liu et al.^30^ analyzed the association between *IL-17* genetic variants and susceptibility to human diseases. They identified significant associations between 18 human diseases and seven genetic variants in *IL-17*. The strongest evidence was between *IL-17A rs2275913, IL-17A rs8193037, IL-17F rs1889570, IL-17F rs763780* and susceptibility to lung and cervical cancer, spondylarthritis, asthma, MS, RA. The bioinformatics analysis suggested that these variants may be associated with or fall in putative functional regions. The explanation for such association may be due to increased expression of proinflammatory cytokines, chemokines, and growth factors. Additionally, *IL-17F* rs763780 SNP could trigger high IL-17 expression which increases the risk of the above-mentioned human diseases.

In the present study, the *IL-17F* rs763780 CT, CC genotypes and C allele were significantly higher in MS patients especially the females and TT genotype and T allele were higher in the healthy controls. These data suggest that the C allele and C containing genotypes may be susceptibility risk factors for the development of MS and the T allele and TT genotype may be protective factors. At the same time, the heterozygous CT genotype was associated with presence of optic neuritis and smoking, longer duration of disease and higher frequency of attacks can be considered as predictor of higher EDSS score.

Atya et al.^31^ and El Sharkawi et al.^32^ work on Egyptian reported a significant increased frequency of *IL-17F* rs763780 CT+CC genotypes and C allele in MS patients and between CT genotype and the delay in disease onset. In contrast, non-significant associations were found between the studied SNP and progression index (PI), or total number of relapses. They also reported an increased frequency of the CT genotype in female patients. This observation was concordant with reported predominance of most autoimmune diseases in female’s especially autoimmune disorders of CNS. This can be attributed to hormonal effects, genetic factors, and exposure to different environmental factors which may explain the differences between male and female as regard the immune system, CNS, and response to immunotherapy^33^.

On contrary, Wang et al.^16^ reported a significantly increased frequency of rs763780 TT genotype and T allele in Chinese Han patients MS patients. The difference between the results of the present study and those of Chinese Han population can be explained by; different sample size; different genotyping method (Real time-PCR vs. RFLP); different environmental exposure; different ethnic background. Manni et al.^34^ in their study on the Y-chromosome gene pool in the modern Egyptian population confirmed the mixture of European, Middle Eastern and African genetic characteristics in Egyptians genetics. More specifically, analysis of Y-chromosome haplotypes demonstrated different distribution of different haplotype between Upper Egypt and Lower Egypt^35^, i.e., different ethnic origins of both populations^36^.

The present study showed a significant association between the heterozygous CT genotype and optic neuritis. The progressive course of MS may lead to more inflammation and damage of the optic nerve. Henderson et al.^37^ used optical coherence tomography (OCT), giving an idea of axonal loss by measuring of the retinal nerve fiber layer (RNFL) and macular volume. They found a significant decrease in the mean of RNFL thickness and macular volume in SPMS group when compared with controls. Tian et al.^38^ investigated IL-17 level and gene expression in the optic nerve to explore the role of Th17 in the pathogenesis of optic neuritis in EAE in mice. They found the concentrations of IL-17 protein in the optic nerve were significantly up-regulated at 11 days post-immunization and mRNA expression of IL-17 was consistent with their protein expression. They indicated that IL-17 may mediate inflammatory pathogenicity at the early stage of optic neuritis. Tian et al.^38^ findings could explain the association between *IL-17F* rs763780 and optic neuritis in the studied Egyptian MS patients. The increased expression and serum level of IL-17 in optic nerve may be involved in the immune-inflammatory pathogenesis of optic neuritis.

Univariable and multivariable regression analysis showed higher scores of EDSS in MS smoker’s patients and this was concordant with findings in many epidemiological studies. Hernan et al.^39^ reported that smoking increases the risk of conversion from RRMS course to SPMS course***.*** They estimated that the risk to be higher three times or more in smokers. Also, Sundstrom and Nystrom^40^ estimated the effect of smoking on the risk of MS progression on newly diagnosed MS cases. They found that smokers were more likely to have progressive disease, and early onset of progression was higher with patients starting smoking before age 15 years. Therefore, cigarette smoking may transform, or accelerate the transformation of the disease into progressive forms. The association can be explained by the increased free radicles and nitric oxide in CNS^41^ and chronic cyanide intoxication leading to widespread demyelination^39^.

## Conclusion

It can be concluded that the IL-17F rs763780 C containing genotypes (CT and CC) and C allele may be risk factors for the development of MS in the studied Egyptian cohort by a gender-dependent mechanism that contributes to tendency for predisposition in females and optic neuritis is more common in patients carrying the CT heterozygous genotype.

## Data Availability

The data underlying this article will be shared on reasonable request to the corresponding author.

## Abbreviations

SNP: Single nucleotide polymorphism
IL: Interleukin
MS: Multiple sclerosis
EBV: Epstein–Barr virus
IMSGC: International Multiple Sclerosis Genetics Consortium
MRI: Magnetic resonance imaging
RRMS: Relapsing–remitting MS
SPMS: Secondary progressive MS
PPMS: Primary progressive MS
PRMS: Progressive-relapsing MS
EDSS: Expanded disability status scale
OCT: Optical coherence tomography
RNFL: Retinal nerve fiber layer
